# Breast Radiologists’ Perceptions on the Detection and Management of Invasive Lobular Carcinoma: Most Agree Imaging Beyond Mammography Is Warranted

**DOI:** 10.1093/jbi/wbad112

**Published:** 2024-02-10

**Authors:** Kristen Coffey, Wendie A Berg, Katerina Dodelzon, Maxine S Jochelson, Lisa A Mullen, Jay R Parikh, Laurie Hutcheson, Lars J Grimm

**Affiliations:** Department of Radiology, Weill Cornell Medicine, New York, NY, USA; Department of Radiology, University of Pittsburgh, Pittsburgh, PA, USA; Department of Radiology, Weill Cornell Medicine, New York, NY, USA; Department of Radiology, Memorial Sloan Kettering Cancer Center, New York, NY, USA; Russell H. Morgan Department of Radiology and Radiological Science, Johns Hopkins Medicine, Baltimore, MD, USA; Division of Diagnostic Imaging, Department of Radiology, The University of Texas MD Anderson Cancer Center, Houston, TX, USA; Lobular Breast Cancer Alliance Inc., White Horse Beach, MA, USA; Department of Radiology, Duke University, Durham, NC, USA

**Keywords:** breast density, breast imaging education and training, mammography including tomosynthesis, contrast-enhanced mammography, breast MRI clinical applications, breast ultrasound

## Abstract

**Objective:**

To determine breast radiologists’ confidence in detecting invasive lobular carcinoma (ILC) on mammography and the perceived need for additional imaging in screening and preoperative settings.

**Methods:**

A 16-item anonymized survey was developed, and IRB exemption obtained, by the Society of Breast Imaging (SBI) Patient Care and Delivery Committee and the Lobular Breast Cancer Alliance. The survey was emailed to 2946 radiologist SBI members on February 15, 2023. The survey recorded demographics, perceived modality-specific sensitivity for ILC to the nearest decile, and opinions on diagnosing ILC in screening and staging imaging. Five-point Likert scales were used (1 = strongly disagree, 2 = disagree, 3 = neutral, 4 = agree, 5 = strongly agree).

**Results:**

Response rate was 12.4% (366/2946). Perceived median (interquartile range) modality-specific sensitivities for ILC were MRI 90% (80–90), contrast-enhanced mammography 80% (70–90), molecular breast imaging 80% (60–90), digital breast tomosynthesis 70% (60–80), US 60% (50–80), and 2D mammography 50% (30–60). Only 25% (85/340) respondents were confident in detecting ILC on screening mammography in dense breasts, while 67% (229/343) were confident if breasts were nondense. Most agreed that supplemental screening is needed to detect ILC in women with dense breasts (272/344, 79%) or a personal history of ILC (248/341, 73%), with 34% (118/334) indicating that supplemental screening would also benefit women with nondense breasts. Most agreed that additional imaging is needed to evaluate extent of disease in women with newly diagnosed ILC, regardless of breast density (dense 320/329, 97%; nondense 263/329, 80%).

**Conclusion:**

Most breast radiologists felt that additional imaging beyond mammography is needed to more confidently screen for and stage ILC.

**Key Messages**

- Breast radiologist survey respondents’ perceived sensitivities for detecting ILC were highest for functional imaging (MRI 90%, contrast-enhanced mammography 80%, molecular breast imaging 80%) and lowest for conventional imaging (digital breast tomosynthesis 70%, US 60%, 2D mammography 50%).
- Most respondents agreed that supplemental screening is needed beyond mammography for women with a personal history of ILC (248/341, 73%) or dense breasts (272/344, 79%).
- Most respondents agreed that additional imaging is needed beyond mammography to evaluate the extent of disease in women with newly diagnosed ILC regardless of breast density (dense breasts 320/329, 97%; non-dense breasts 263/329, 80%).

## Introduction

Invasive lobular carcinoma (ILC) is the second most common type of invasive breast cancer, accounting for 15% of all breast cancers, and is recognized by its unique pathologic, imaging, and clinical features (1–3). Invasive lobular breast cancer is characterized by loss of E-cadherin and its distinctive single-file growth pattern that may result in minimal disruption of surrounding tissue architecture (4). The absence of a desmoplastic response is a salient feature of some ILC presentations that enables disease progression with relatively subtle imaging and clinical findings. Invasive lobular breast cancer has a higher propensity than invasive ductal carcinoma (IDC) to have progressed to locally advanced, multifocal, multicentric, bilateral, regional nodal, or metastatic disease by time of diagnosis (2,5–7).

While mammography is the standard for breast cancer screening and is the first-line modality for most diagnostic breast evaluations, its sensitivity relies on the detection of morphologic changes that may not be evident with ILC (3). Mammographically, ILC typically presents as a mass or with subtle findings, such as architectural distortion or a low-density asymmetry that may only be seen in one view in up to 35% of cases (6,8–13). Calcifications are infrequent, with a prevalence of 0% to 24% in the literature (14). Noncalcified cancers of any type are often masked by dense breast tissue, with mammographic sensitivity inversely related to breast density (15,16). Reduced mammographic performance has been reported particularly in women with ILC and dense breasts (12). Additionally, 2D mammography has demonstrated significantly lower sensitivity for ILC compared with IDC, irrespective of breast density (9). The combination of both dense breast tissue and subtle imaging findings likely contributes to the high false-negative rate of ILC on mammography, reaching 43% in one older published series using 2D mammography (8).

In the early 2000s, digital mammography replaced film-screen mammography with no significant improvement in sensitivity for ILC (6). Since then, newer mammographic techniques such as digital breast tomosynthesis (DBT) and contrast-enhanced mammography (CEM), which are quasi-3D and dual-energy vascular-based techniques, respectively, have shown improved detection of ILC in a few small studies (17,18). Although the sonographic appearance of ILC can be variable and nonspecific, US is not limited by breast density and may be a valuable adjunct in detecting mammographically occult ILC (12–14). Contrast-enhanced MRI has the highest sensitivity for ILC and is valuable in defining extent of disease in women with ILC (3,12,19,20). However, its use as a screening tool is currently limited to certain groups of women who are at an elevated risk for breast cancer (21). Molecular breast imaging (MBI) is another functional technique that captures intravenous technetium-99m sestamibi uptake in the breast with early data showing its potential as a mammography supplement (3,22,23).

The purpose of our study was to survey a large community of breast radiologists from different practice settings, and with different training and experience, to better understand perceptions of how to best diagnose and subsequently manage ILC with imaging. First, we assessed perceived sensitivity of various imaging modalities for detecting ILC. Second, we assessed their confidence interpreting mammography to diagnose ILC and need for additional imaging in both screening and preoperative settings, with a secondary focus on dense versus non-dense breasts. We hypothesized that our survey results would show that most radiologists deem functional imaging such as MRI or CEM to be superior to conventional mammography for detecting ILC, which might further support and improve access to these techniques for supplemental screening and staging.

## Methods

### Survey development

A 16-item survey was developed by the Society of Breast Imaging (SBI) Patient Care and Delivery (PC&D) committee in partnership with the Lobular Breast Cancer Alliance (LBCA) to assess radiologists’ confidence in diagnosing ILC on mammography and other breast imaging modalities. The survey was approved as exempt by the Institutional Review Board of Duke University. All respondents were first asked to rate the sensitivity of different imaging modalities for detecting ILC based on personal experience from 1% to 100% (to the nearest 10%). The survey was then divided into 2 sections addressing different clinical scenarios: *screening* and *staging*. Screening included screening of the general population as well as surveillance of women with previously treated ILC. Staging was defined as diagnostic imaging performed to evaluate extent of disease within the breast in women with newly diagnosed ILC. Similar questions using 5-point Likert scale responses were asked in parallel in both screening and staging sections to assess respondents’ confidence using mammography and additional imaging in each setting. Some questions were split with separate answers for dense and nondense breasts, using Breast Imaging Reporting and Data System (BI-RADS) breast density categories (A and B for nondense, C and D for dense) (24). The survey questions focused on diagnosing ILC in women because male ILC is exceptionally rare, comprising only 1% of all male breast cancers (25).

Demographic information collected included practice type, breast imaging fellowship status, years in practice, and percentage of clinical time devoted to breast imaging. For the subanalyses, fellowship training included programs less than, equal to, or greater than 6 months in duration, and nonfellowship training also included training before breast imaging fellowships existed. Responses for years in practice and percentage of clinical time devoted to breast imaging were reported on a sliding scale, with median values used for sub-analyses calculations.

The survey was initially developed by a core group of 7 breast radiologists (6 from the SBI PC&D committee and 1 from the LBCA) and 1 nonradiologist who serves as the LBCA Executive Director. The survey was piloted at 4 institutions to solicit feedback and refine the organization, language, and content of the survey. The pilot data were not included in the analysis. The final version of the administered survey can be found in the online supplementary material (Table S1).

### Survey administration

The survey was sent electronically to all SBI board-eligible/certified radiologist members in the U.S. or Canada via email on February 15, 2023, from the SBI. Members-in-training were not included. The email contained an electronic link to the survey hosted on SurveyMonkey (San Mateo, CA). Three reminder emails were sent at approximately 2-week intervals, and data were collected for 8 weeks. Questions could be skipped, and partial surveys could be submitted. All survey responses were voluntary and anonymized.

### Data analysis

Median scores with interquartile range (IQR) were calculated for the sensitivity responses. Comparisons between sensitivities were made using Welch’s two-sample *t* test. Comparisons between sensitivity and demographic variables were made using Kruskal–Wallis rank sum test and Wilcoxon rank sum test as appropriate. The remaining responses were scored using a 5-point Likert scale and median scores calculated (Strongly Agree = 5, Agree = 4, Neither Agree nor Disagree = 3, Disagree = 2, Strongly Disagree = 1). Odds ratios with CIs were calculated to measure association between Likert scores and demographic variables. A *P*-value of .05 or less was considered statistically significant. Statistical analysis was performed using R.2 (R Foundation for Statistical Computing, Vienna, Austria).

## Results

### Demographics

The survey invitation was delivered to 2946 members and opened by 2005 recipients (Table S1). A total of 366 participants completed at least some component of the survey for a response rate of 12.4%. Of 366 respondents, 303 (83%) completed the entire survey, whereas 63/366 (17%) partially completed the survey.

**Table 1** shows the demographic characteristics of the 366 radiologist members of the SBI who responded to the survey. The majority of respondents work in either academic (113/331, 34%) or private practice (145/331, 44%) settings, are breast fellowship trained (239/331, 72%), and devote 100% of clinical time to breast imaging (189/330, 57%). The median number of years in practice was 16.5 (IQR 9.25, 29.0). Most radiologists are currently offering either DBT (326/366, 89%) or 2D mammography (268/366, 73%), US (329/366, 90%), and MRI (313/366, 86%) in their practice, while CEM and MBI are currently being offered by only 19% (69/366) and 7% (27/366), respectively. A plurality of respondents (158/366, 43%) are not planning to offer any new imaging services in the next 2 years, followed by 26% (94/366) who are planning on adding CEM. Additional free text responses to other current or future services included automated whole breast US, abbreviated MRI, cone beam breast CT, and interventional procedures such as high-intensity focused US and CEM-guided biopsy.

**Table 1.** Demographic Information for Respondents (*N* = 366)

Demographic variable	*n* (%) or median (IQR)
*Practice type*	
No. of responses	331 (90)
Academic	113 (34)
Private	145 (44)
Academic/private hybrid	54 (16)
Governmental/Veterans Affairs	7 (2.1)
Other	12 (3.6)
No. missing	35 (9.6)
*Breast fellowship status*	
No. of responses	331 (90)
Fellowship trained (≤6 months)	43 (13)
Fellowship trained (>6 months)	196 (59)
Not fellowship trained	28 (8.5)
Trained before fellowships existed	64 (19)
No. missing	35 (9.6)
*Years in practice*	
No. of responses	330 (90)
Years in practice	16.5 (9.3–29)
No. missing	36 (9.8)
*Percent time in breast imaging*	
No. of responses	330 (90)
Percent time in breast imaging	100 (80–100)
No. missing	36 (9.8)
*Currently offered services*	
2D digital mammography	268 (73)
Digital breast tomosynthesis	326 (89)
Breast US	329 (90)
Breast MRI	313 (86)
Contrast-enhanced mammography	69 (19)
Molecular breast imaging	27 (7.4)
Other	16 (4.4)^a^

Abbreviation: IQR, interquartile range.

^a^Free text responses included automatic breast US (8), cone beam computed tomography (3), high-intensity focused US, biopsies/localizations, independent contractor, retired, and focused physical examination.

### Modality sensitivities

**Table 2** depicts responses for the question, “In your experience, how sensitive is each of the following modalities for detecting IL*C*?” 2D mammography had the lowest estimated mean sensitivity of 50% (IQR 30–60), which was significantly inferior to all other modalities (all *P* <.001). MRI had the highest estimated sensitivity of 90% (IQR 80–90). DBT (70%) was rated as significantly more sensitive than 2D mammography (50%, *P* <.001) or US (60%, *P* = .003) but significantly less sensitive than MRI (90%, *P* <.001), CEM (80%, *P* <.001), or MBI (80%, *P* = .016). Of note, a majority of respondents reported being unsure of CEM and MBI sensitivities (232/366, 63% and 282/366, 77%, respectively).

**Table 2.** Respondents’ Perception of Modality-Specific Sensitivity for Detecting ILC (*N* = 366)

Modality	Missing, *n* (%)	Unsure, *n* (%)	Responded, *n* (%)	Estimated median sensitivity %^a^ (IQR)	Compared to 2D DM^b^	Compared to DBT^b^
2D DM	0	20 (5.5)	346 (95)	50 (30–60)	-	*P* <.001^*^
DBT	2 (0.6)	16 (4.4)	348 (95)	70 (60–80)	*P* <.001^*^	-
US	2 (0.6)	18 (4.9)	346 (95)	60 (50–80)	*P* <.001^*^	*P* = . 003^*^
MRI	1 (0.3)	10 (2.7)	355 (97)	90 (80–90)	*P* <.001^*^	*P* <.001^*^
CEM	4 (1.1)	232 (63)	130 (36)	80 (70–90)	*P* <.001^*^	*P* <.001^*^
MBI	4 (1.1)	282 (77)	80 (22)	80 (60–90)	*P* <.001^*^	*P* = . 016^*^

Abbreviations: CEM, contrast-enhanced mammography; DBT, digital breast tomosynthesis; DM, digital mammography; ILC, invasive lobular carcinoma; IQR, interquartile range; MBI, molecular breast imaging.

^*^Statistically significant.

^a^Response options were provided in 10% increments.

^b^Welch’s two-sample *t* test was used.

Respondents with 16.5 years or more of experience (median) perceived 2D mammography as slightly more sensitive than those with less experience (50% vs 40%, *P* = .042). The sensitivity of CEM was perceived higher among academic radiologists than those in private or hybrid practice settings (90% vs 85% and 80%, respectively, *P* = .057). No other demographic variable was significantly associated with the sensitivity estimates. Table S2 shows comparison between modality-specific sensitivities and demographic variables.

### Screening

**Figure 1.**
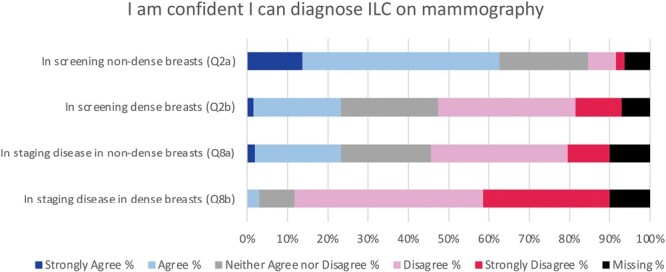
Likert responses to questions assessing 366 breast imaging radiologists’ confidence in diagnosing invasive lobular carcinoma (ILC) on mammography (2D or digital breast tomosynthesis) in various clinical scenarios.

Most respondents agreed (229/343, 66.8%) with feeling confident diagnosing ILC on screening mammography/DBT if the breasts are nondense (median 4) (**Figure 1**, Table S3). For dense breasts, however, confidence levels were mixed (median 3) as nearly one-half of respondents disagreed with feeling confident (166/340, 48.8%) and the other half were split between feeling confident (85/340, 25.0%) or neutral (89/340, 26.2%).

Most respondents agreed (319/345, 92.5%) that their confidence in diagnosing ILC on screening mammography/DBT is lower in women with dense breasts compared with nondense breasts (median 4). Opinion was divided (median 3) as to whether perceived confidence is lower in women with a history of treated ILC, with 40.0% (138/345) agreeing, 40.6% (140/345) neither agreeing nor disagreeing, and 19.4% (67/345) disagreeing.

**Figure 2.**
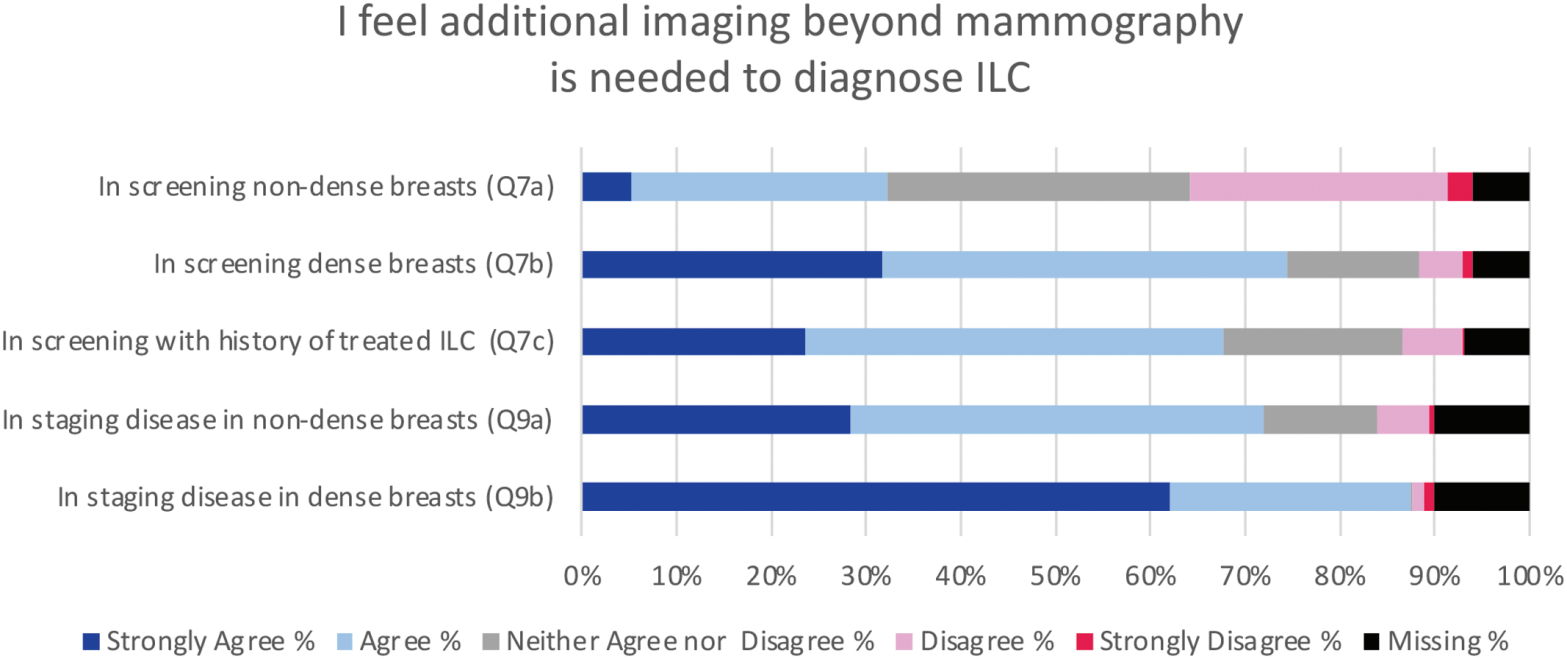
Likert responses to questions assessing 366 breast imaging radiologists’ opinion on whether they need additional imaging to diagnose invasive lobular carcinoma (ILC) on mammography (2D or digital breast tomosynthesis) in various clinical scenarios.

Most respondents agreed (median 4) that additional screening beyond mammography/DBT is needed to diagnose ILC in women who have dense breasts (272/344, 79.1%) or a history of prior treated ILC (248/341, 72.7%) (**Figure 2**). However, there was no consensus (median 3) as to whether additional screening is needed for women with nondense breasts, with 34.3% (118/344) agreeing, 31.7% (109/344) disagreeing, and 34.0% (117/344) neither agreeing nor disagreeing.

When reading a screening mammogram in a woman with a history of breast cancer, most respondents agreed (median 4) that knowledge of prior histopathology is helpful (79.4%, 273/344) and usually available to them (65.0%, 223/343) during interpretation.

Compared to fellowship-trained radiologists, radiologists without fellowship training were significantly more likely to disagree that they are confident in diagnosing ILC on screening mammography in both dense (odds ratio [OR] 2.32, 95% confidence interval [CI]: 1.49–3.64, *P* <.001) and nondense breasts (OR 1.59, 95% CI: 1.01–2.52, *P* = .046) and less likely to disagree that their confidence is lower with a history of prior ILC (OR 0.63, 95% CI: 0.41–0.97, *P* = .035) (Table S4). Non–fellowship-trained radiologists were also significantly less likely to disagree that additional screening beyond mammography is needed to detect ILC in women with dense breasts (OR 0.53, 95% CI: 0.34–0.84, *P* = .006).

### Staging

A small minority (11/329, 3.3%) of respondents agreed with feeling confident in evaluating extent of ILC disease on mammography/DBT in women with dense breasts, and no respondent chose “strongly agree” (median 2) (**Figure 1**, Table S3). For nondense breasts, however, opinion was divided (median 3) as nearly one-half of respondents (162/329, 49.2%) disagreed with feeling confident and the other half were split between feeling confident (85/329, 25.8%) or neutral (82/329, 24.9%).

The overwhelming majority of respondents agreed that additional imaging beyond mammography/DBT is needed to evaluate extent of ILC disease in both women with dense breasts (320/329, 97.3%) (median 5) and nondense breasts (263/329, 79.9%) (median 4) (**Figure 2**). There was strong agreement (median 5) that respondents routinely recommend MRI to evaluate extent of ILC disease in women with dense breasts (307/323, 95.1%) as well as nondense breasts (262/325, 80.6%).

Radiologists with fellowship training were significantly more likely to agree that they routinely recommend preoperative MRI in women with dense breasts (OR 1.81, 95% CI: 1.03–3.15, *P* = .041) compared with those who are not fellowship trained (Table S4). Similarly, radiologists who devote 100% time in breast were also more likely to recommend MRI regardless of breast density (OR 1.69, 95% CI: 0.99–2.89, *P* = .056 for dense and OR 1.72, 95% CI, 1.13–2.61, *P* = .011 for nondense).

## Discussion

Our study provides insights into breast radiologists’ experience-based perceptions of the diagnostic accuracy of current imaging tools for screening and staging ILC. The first part of the survey revealed radiologists’ perception that conventional imaging modalities (2D mammography, DBT, US) are less sensitive for detecting ILC compared with newer functional techniques (MRI, CEM, MBI). Specifically, the pooled sensitivity of 2D mammography was estimated at only 50%, significantly lower than all other modalities (*P* < .001), consistent with prior literature data (sensitivity range 34%–81%) (3,5,6,12,17,26). Our finding that both DBT and US are perceived to be significantly more sensitive than 2D mammography (*P* <.001 and *P* = .003, respectively) confirms prior research showing superior performance of both modalities in the detection of ILC (11–13,17,26,27). In the literature, DBT has demonstrated improved mammographic accuracy and sensitivity (up to 85%) for detecting ILC and specifically for tumors presenting as architectural distortion or masses (17,28). Similarly, most studies have shown that US outperforms 2D mammography in detecting ILC with higher sensitivity (up to 98%), including one series (13) in which US identified 88% of mammographically occult ILC (11,12,26,27). Interestingly, we found that radiologists perceive DBT to be significantly more sensitive than US (estimated at 70% vs 60%, *P* = .003), and the perceived sensitivity of US (average 60% in our survey) was lower than actual sensitivity reported in literature (range 68%–98%) (29). These findings may reflect bias against use of US for screening given the relatively low incremental cancer detection rates observed after 2D mammography (2–3 per 1000) and tomosynthesis (1.1 per 1000) as well as the relatively low positive predictive value of biopsies prompted only by screening US (9%–11%) (30,31).

MRI was rated the most sensitive modality for diagnosing ILC (90%), which is consistent with prior studies (sensitivity 93%–100%) predominantly performed in the preoperative setting (3,12,20,22,32). Abbreviated MRI protocols mitigate the high cost that has traditionally limited widespread implementation of screening MRI and might lead to earlier detection of breast cancers, including ILC (33,34). Contrast-enhanced mammography and MBI are 2 newer functional techniques that were both perceived to be highly sensitive (80%) by surveyed radiologists and may be promising alternatives to MRI. Contrast-enhanced mammography is potentially more cost-efficient than MRI, supporting our finding that one-quarter (26%) of respondents plan to start offering CEM within the next 2 years (35). The sensitivity of CEM was estimated to be higher by academic radiologists (*P* = .057), likely reflecting greater awareness of preliminary data and the early adoption of CEM at some academic centers. Contrast-enhanced mammography is a promising alternative to MRI for both screening and diagnostic evaluations, with data from 3 meta-analyses yielding a pooled sensitivity and specificity of 95% to 97% and 66% to 81% for all cancers, respectively (36–38). While CEM has been shown to outperform 2D mammography for staging new ILC (sensitivity up to 100%), CEM was slightly less sensitive (78%–88%) but more specific (92%–99%) than MRI for detecting additional sites of disease in a few studies (18,39–41). The sensitivity of MBI for detecting ILC varies in the literature, ranging from 57% to 93% (22,23,42). Molecular breast imaging was significantly less sensitive for ILC compared with IDC (57% vs 86%, *P* <.001) in one large study and has demonstrated variable performance compared to MRI in smaller cohorts (18,22,23). More than one-half (63%) and three-quarters (77%) of respondents were unsure about CEM and MBI sensitivities, respectively, underscoring the need for more widespread validation of these modalities in the screening setting.

Only one-quarter (85/340) of surveyed radiologists were confident in their ability to diagnose ILC on screening mammography in women with dense breasts, with most agreeing (272/344, 79%) that supplemental screening is needed for these women. This is not a surprising result because higher breast density reduces the sensitivity of mammography for detecting all types of breast cancer, and ILC often presents with subtle and usually noncalcified mammographic findings (9,12). Recently updated American College of Radiology (ACR) guidelines recommend that women with dense breasts who desire supplemental screening have MRI; for eligible women who cannot access or tolerate MRI, CEM is recommended, or US if contrast-based methods are unavailable (21). Most respondents (248/341, 73%) also felt that supplemental screening would benefit women with a personal history of ILC, with 40% (138/345) reporting lower confidence in their mammographic interpretation if a personal history was present. These findings are important because supplemental screening MRI is currently recommended for women with a personal history of breast cancer if they also have dense breasts or if they were diagnosed before age 50 (21). Women with a personal history of ILC diagnosed after age 50 or with nondense breasts should consider MRI if supplemental screening is desired, and our survey respondents agreed with this approach (43). For women with non-dense breasts, more than half of radiologists (229/343, 67%) felt confident in their ability to diagnose ILC on screening mammography. Yet, approximately one-third (118/344, 34%) also felt that supplemental screening was still needed for these women and one-third (117/334, 34%) were unsure. Currently, women with nondense breasts are only recommended to undergo supplemental screening if breast cancer risk factors are present.

A majority of radiologists in our survey routinely recommend preoperative MRI for staging disease in women with newly diagnosed ILC, regardless of breast density (307/323, 95% dense and 262/325, 81% nondense). Nearly all radiologists (320/329, 97%) reported a lack of confidence in assessing extent of ILC disease on mammography in dense breasts, and nearly half (162/329, 49%) felt similarly if breasts were nondense. Preoperative MRI for staging ILC is currently recommended by several guidelines in the United States and Europe, including the ACR (2018), National Institute for Health and Care Excellence (2018), American Society of Breast Surgeons (2017), European Society of Breast Imaging (2008), European Society of Breast Cancer Specialists (2010), and German Gynecological Oncology Group (2018) (19). The National Comprehensive Cancer Network (2023) recommendation for preoperative MRI is optional for evaluating the extent of ILC that is poorly defined on mammography, US, or physical examination (44). These guidelines are supported by prior research demonstrating superior performance of preoperative MRI compared with mammography for staging ILC disease (12,45–47). One meta-analysis found that MRI detected additional sites of ipsilateral and contralateral disease in 32% and 7% of patients with ILC, respectively, that were occult on mammography and/or US (46). Another more recent meta-analysis found that preoperative MRI did not improve surgical outcomes for patients with ILC, and therefore, the practice remains somewhat controversial (48).

A few significant trends were observed between demographic variables and responses. Greater clinical experience (≥16.5 years in practice) was associated with higher perceived sensitivity of 2D mammography, which may suggest that radiologists who have been in practice longer are more confident interpreting 2D mammography compared to those who were also trained in DBT and/or CEM. Another statistically significant trend was that, compared with fellowship-trained radiologists, those without fellowship training were more likely to report lower confidence in detecting ILC on screening mammography regardless of breast density, to report lower confidence in their mammographic interpretation if there was a history of prior ILC, and to want additional screening in women with dense breasts. A final observation was that radiologists who devote 100% of their clinical time to breast (compared with <100% time) were more likely to want additional preoperative imaging in women with non-dense breasts and to routinely recommend MRI for assessing disease extent not only in dense breasts but also in nondense breasts. It is possible that radiologists who interpret 100% breast imaging also interpret a higher volume of preoperative MRIs and, based on this experience, understand the benefit of performing MRI across breast density categories.

This study has a few limitations. First, all respondents are SBI members who are predominantly breast imaging subspecialists, either by training or experience, and therefore the opinions expressed may not be generalizable to the entire radiology community. Similarly, with a response rate of 12%, results may not be applicable to the entire SBI membership. Second, there seemed to be less consensus among responses to questions about “confidence” compared with questions about “need for additional imaging.” For example, there was no consensus (median 3 = neutral) for the questions asking about confidence interpreting mammography with dense breasts or history of prior ILC. Yet, most radiologists later agreed that supplemental screening is needed for both groups. This observation that “confidence” responses were more divided than the “need for additional imaging” questions may reflect a difference in language, where “confidence” may be a more subjective term compared with the more absolute term “need.” Third, the percentage of clinical time devoted to breast imaging may have changed over the course of a radiologist’s career, and therefore, the current percentage may not accurately reflect previous experience. Lastly, several responses to the final question about future services not currently offered included 2D mammography, indicating that this question was misinterpreted, limiting analysis of this question. Though not specifically evaluated in this survey, a few free text responses to this question included artificial intelligence as a future service, which has been shown to improve sensitivity of ILC detection on mammography and US and warrants further study (49,50).

Overall, surveyed breast radiologists perceived 2D mammography to be the least sensitive and MRI to be the most sensitive modalities for ILC detection. Although the majority of radiologists were unsure about CEM and MBI sensitivities, the perceived estimated sensitivities for these emerging functional techniques were high. Most radiologists agreed that additional imaging beyond mammography is warranted to screen for ILC in women with dense breasts and to evaluate extent of disease in women with newly diagnosed ILC regardless of breast density, in accordance with current guidelines. For women with a personal history of ILC, most radiologists agreed supplemental screening was needed, a finding that should encourage those with nondense breasts or those diagnosed after age 50 to strongly consider annual MRI (21).

## Conclusion

Our survey results confirm the low sensitivity of mammography for ILC as perceived by breast radiologists across different practice settings, training, and experience and should support future educational, research, and clinical initiatives to raise awareness of the challenges in diagnosing ILC on conventional imaging and advocate for functional techniques that might improve earlier detection of this sometimes insidious disease.

## Supplementary Material

wbad112_suppl_Supplementary_Tables_S1-S4
