# Supplementary material for: Breast Radiologists’ Perceptions on the Detection and Management of Invasive Lobular Carcinoma: Most Agree Imaging Beyond Mammography Is Warranted
Source: J Breast Imaging. 2024 Feb 10;6(2):157–65. doi: 10.1093/jbi/wbad112 (PMC10983784; doi:10.1093/jbi/wbad112)
Supplement: wbad112_suppl_Supplementary_Tables_S1-S4 [file wbad112_suppl_supplementary_tables_s1-s4.docx]

**Supplementary Material**

**Table S1.** Society of Breast Imaging Survey Email Invitation Data

| **Date of email** | **Delivered, N** | **Unique open,^a^ n (%)** | **Unique click,^b^ n (%)** |
| --- | --- | --- | --- |
| 2/15/23 | 2915 | 2005 (68.9) | 157 (7.8) |
| 2/21/23 | 2920 | 1998 (68.5) | 134 (6.7) |
| 3/9/23 | 2931 | 1907 (65.1) | 156 (8.2) |
| 3/24/23 | 2946 | 1895 (64.3) | 113 (6) |

^a^Number of contacts who opened the email.

^b^Number of contacts who clicked on survey link.

**Table S2.** Modality-Specific Estimated Sensitivity Compared with Demographic Variables (N = 366)

| **Demographic variable** | **Missing, n (%)** | **Responded, n (%)** | **Estimated modality sensitivity to nearest 10%, median (IQR)** | | | | | |
| --- | --- | --- | --- | --- | --- | --- | --- | --- |
|  |  |  | **2D DM** | **DBT** | **US** | **MRI** | **CEM** | **MBI** |
| Practice type | 54 (14.8)^a^ | 312 (85.2) |  |  |  |  |  |  |
|  |  | Academic N = 113 | 0.50 (0.30, 0.60) | 0.70 (0.50, 0.80) | 0.60 (0.50, 0.80) | 0.90 (0.80, 0.90) | 0.90 (0.70, 0.90) | 0.80 (0.70, 0.83) |
|  |  | Private N = 145 | 0.50 (0.30, 0.60) | 0.70 (0.60, 0.80) | 0.70 (0.50, 0.80) | 0.90 (0.80, 0.90) | 0.85 (0.80, 0.90) | 0.80 (0.70, 0.88) |
|  |  | Hybrid N = 54 | 0.40 (0.30, 0.50) | 0.70 (0.50, 0.80) | 0.60 (0.50, 0.78) | 0.90 (0.80, 0.90) | 0.80 (0.70, 0.80) | 0.70 (0.60, 0.88) |
|  |  | *P*-value^b^ | 0.2 | 0.4 | 0.6 | 0.6 | 0.057 | 0.7 |
| Fellowship status | 35 (9.6) | 331 (90.4) |  |  |  |  |  |  |
|  |  | Trained N = 239 | 0.50 (0.30, 0.60) | 0.70 (0.60, 0.80) | 0.60 (0.50, 0.80) | 0.90 (0.80, 0.90) | 0.80 (0.70, 0.90) | 0.80 (0.60, 0.90) |
|  |  | Not trained N = 92 | 0.50 (0.30, 0.60) | 0.70 (0.60, 0.80) | 0.60 (0.50, 0.80) | 0.90 (0.80, 0.90) | 0.80 (0.70, 0.90) | 0.80 (0.70, 0.80) |
|  |  | *P*-value^c^ | 0.4 | >0.9 | 0.6 | 0.9 | >0.9 | 0.7 |
| Years in practice | 36 (9.8) | 330 (90.2) |  |  |  |  |  |  |
|  |  | <16.5 years N = 165 | 0.40 (0.30, 0.50) | 0.70 (0.50, 0.80) | 0.60 (0.50, 0.70) | 0.90 (0.80, 0.90) | 0.80 (0.70, 0.90) | 0.80 (0.60, 0.80) |
|  |  | ≥16.5 years N = 165 | 0.50 (0.30, 0.60) | 0.70 (0.60, 0.80) | 0.60 (0.50, 0.80) | 0.90 (0.80, 0.90) | 0.80 (0.70, 0.90) | 0.80 (0.70, 0.90) |
|  |  | *P*-value^c^ | 0.042* | 0.8 | 0.2 | 0.8 | 0.8 | 0.2 |
| Percent time in breast | 36 (9.8) | 330 (90.2) |  |  |  |  |  |  |
|  |  | <100% time N = 141 | 0.40 (0.30, 0.50) | 0.70 (0.50, 0.75) | 0.60 (0.50, 0.80) | 0.90 (0.80, 0.90) | 0.80 (0.70, 0.90) | 0.80 (0.60, 0.80) |
|  |  | 100% time N = 189 | 0.50 (0.30, 0.60) | 0.70 (0.60, 0.80) | 0.60 (0.50, 0.80) | 0.90 (0.80, 0.90) | 0.80 (0.80, 0.90) | 0.80 (0.68, 0.90) |
|  |  | *P*-value^c^ | 0.065 | 0.2 | >0.9 | 0.2 | 0.2 | 0.6 |

Abbreviations: CEM, contrast-enhanced mammography; DBT, digital breast tomosynthesis; DM, digital mammography; IQR, interquartile range; MBI, molecular breast imaging.

*Statistically significant.

^a^Also includes “governmental/veterans affairs” and “other” responses too small to include in analysis.

^b^Kruskal-Wallis rank sum test was used.

^c^Wilcoxon rank sum test was used.

**Table S3.** Likert Scale Responses on a Scale of 1 (Strongly Disagree) to 5 (Strongly Agree) (N = 366)

| **Question** | **Missing responses, n** | **Total responses, n** | **Agree,^a^ n (%)** | **Neither disagree or agree,^b^ n (%)** | **Disagree,^c^ n (%)** | **Median** |
| --- | --- | --- | --- | --- | --- | --- |
| **Screening for ILC** | | | | | | |
| Q2a. I am confident I can diagnose ILC on screening mammography (2D with DBT) in women with **non-dense breasts** | 23 | 343 | 229 (66.7) | 81 (23.6) | 33 (9.6) | 4 |
| Q2b. I am confident I can diagnose ILC on screening mammography (2D with DBT) in women with **dense breasts** | 26 | 340 | 85 (25.0) | 89 (26.2) | 166 (48.8) | 3 |
| Q3. My confidence in diagnosing ILC on screening mammography/DBT is lower in women with dense breasts compared with non-dense breasts | 21 | 345 | 319 (92.5) | 12 (3.5) | 14 (4.1) | 4 |
| Q4. My confidence in diagnosing ILC on screening mammography/DBT is lower in women who have a history of treated ILC | 21 | 345 | 138 (40.0) | 140 (40.6) | 67 (19.4) | 3 |
| Q5. I find it helpful to know the histopathology of any prior breast cancer before reading a screening mammogram | 22 | 344 | 273 (79.4) | 39 (11.4) | 32 (9.3) | 4 |
| Q6. When I read a screening mammogram in a woman with a history of breast cancer, details of histopathology are usually available to me | 23 | 343 | 223 (65.0) | 42 (12.2) | 78 (22.7) | 4 |
| Q7a. I feel additional screening beyond mammography/DBT is needed to diagnose ILC in women with **non-dense breasts** | 22 | 344 | 118 (34.3) | 117 (34.0) | 109 (31.7) | 3 |
| Q7b. I feel additional screening beyond mammography/DBT is needed to diagnose ILC in women with **dense breasts** | 22 | 344 | 272 (79.1) | 51 (14.8) | 21 (6.1) | 4 |
| Q7c. I feel additional screening beyond mammography/DBT is needed to diagnose ILC in women with **history of treated ILC** | 25 | 341 | 248 (72.7) | 69 (20.2) | 24 (7.0) | 4 |
| **Staging ILC** | | | | | | |
| Q8a. I am confident I can evaluate extent of ILC disease on mammography (2D with DBT) in women with **non-dense breasts** | 37 | 329 | 85 (25.8) | 82 (24.9) | 162 (49.2) | 3 |
| Q8b. I am confident I can evaluate extent of ILC disease on mammography (2D with DBT) in women with **dense breasts** | 37 | 329 | 11 (3.3) | 32 (9.7) | 286 (86.9) | 2 |
| Q9a. I feel additional imaging beyond mammography/DBT is needed to evaluate the extent of ILC disease in women with **non-dense breasts** | 37 | 329 | 263 (79.9) | 44 (13.4) | 22 (6.7) | 4 |
| Q9b. I feel additional imaging beyond mammography/DBT is needed to evaluate the extent of ILC disease in women with **dense breasts** | 37 | 329 | 320 (97.3) | 4 (0.3) | 8 (2.4) | 5 |
| Q10a. I routinely recommend MRI to evaluate the extent of ILC disease in women with **non-dense breasts** | 41^d^ | 325 | 262 (80.6) | 46 (14.2) | 17 (5.2) | 5 |
| Q10b. I routinely recommend MRI to evaluate the extent of ILC disease in women with **dense breasts** | 43^a^ | 323 | 307 (95.0) | 11 (3.4) | 5 (1.5) | 5 |

Abbreviations: DBT, digital breast tomosynthesis; DM, digital mammography; ILC, invasive lobular cancer.

^a^Includes responses 4 (agree) or 5 (strongly agree).

^b^Includes response 3 (neither agree nor disagree).

^c^Includes responses 1 (strongly disagree) or 2 (disagree).

^d^Includes response “not applicable.”

**Table S4.** Comparison of Demographic Variables with Likert Responses (Odds Ratios with 95% CI)

| **Question** | **Practice type** | | | | **Fellowship status** | | | **Years in practice** | | | **Percent time in breast** | | |
| --- | --- | --- | --- | --- | --- | --- | --- | --- | --- | --- | --- | --- | --- |
|  | Academic^a^ | Private, OR (95% CI) | Hybrid, OR (95% CI) | *P*-value | Trained, OR (95% CI) | Not trained,^a^ OR (95% CI) | *P*-value | <16.5,^a^ OR (95% CI) | ≥16.5, OR (95% CI) | *P*-value | <100,^a^ OR (95% CI) | 100, OR (95% CI) | *P*-value |
| *Screening* | | | | | | | | | | | | | |
| Q2a. I am confident I can diagnose ILC on screening mammography (2D with DBT) in women with **non-dense breasts** | - | 1.22 (0.76, 1.94) | 1.13 (0.60, 2.12) | 0.7 | 1.59 (1.01, 2.52) | - | 0.046* | - | 0.58 (0.38, 0.87) | 0.009* | - | 1.03 (0.68, 1.56) | 0.9 |
| Q2b. I am confident I can diagnose ILC on screening mammography (2D with DBT) in women with **dense breasts** | - | 1.02 (0.65, 1.60) | 1.2 (0.67, 2.14) | 0.8 | 2.32 (1.49, 3.64) | - | <0.001* | - | 0.70 (0.47, 1.04) | 0.079 | - | 1.71 (1.14, 2.55) | 0.009* |
| Q3. My confidence in diagnosing ILC on screening mammography/DBT is lower in women with dense breasts compared with non-dense breasts | - | 0.69 (0.42, 1.11) | 0.64 (0.34, 1.22) | 0.2 | 1.05 (0.65, 1.67) | - | 0.9 | - | 1.08 (0.71, 1.65) | 0.7 | - | 0.80 (0.52, 1.23) | 0.3 |
| Q4. My confidence in diagnosing ILC on screening mammography/DBT is lower in women who have a history of treated ILC | - | 1.09 (0.69, 1.72) | 0.87 (0.48, 1.57) | 0.7 | 0.63 (0.41, 0.97) | - | 0.035* | - | 1.22 (0.82, 1.82) | 0.3 | - | 0.85 (0.57, 1.27) | 0.4 |
| Q5. I find it helpful to know the histopathology of any prior breast cancer before reading a screening mammogram | - | 0.91 (0.57, 1.45) | 1.02 (0.55, 1.88) | 0.9 | 1.38 (0.88, 2.18) | - | 0.2 | - | 0.79 (0.52, 1.19) | 0.3 | - | 1.55 (1.02, 2.35) | 0.04* |
| Q6. When I read a screening mammogram in a woman with a history of breast cancer, details of histopathology are usually available to me | - | 0.39 (0.24, 0.63) | 0.36 (0.20, 0.67) | <0.001* | 1.80 (1.15, 2.82) | - | 0.01* | - | 0.79 (0.53, 1.18) | 0.3 | - | 1.61 (1.08, 2.43) | 0.02* |
| Q7a. I feel additional screening beyond mammography/DBT is needed to diagnose ILC in women with **non-dense breasts** | - | 0.78 (0.50, 1.22) | 1.09 (0.60, 1.97) | 0.4 | 0.79 (0.51, 1.22) | - | 0.3 | - | 1.56 (1.05, 2.31) | 0.028* | - | 1.28 (0.86, 1.91) | 0.2 |
| Q7b.I feel additional screening beyond mammography/DBT is needed to diagnose ILC in women with **dense breasts** | - | 0.83 (0.52, 1.30) | 0.71 (0.38, 1.33) | 0.5 | 0.53 (0.34, 0.84) | - | 0.006* | - | 1.77 (1.18, 2.67) | 0.006* | - | 1.11 (0.74, 1.67) | 0.6 |
| *Staging* | | | | | | | | | | | | | |
| Q8a. I am confident I can evaluate extent of ILC disease on mammography (2D with DBT) in women with **non-dense breasts** | - | 0.99 (0.63, 1.55) | 1.07 (0.59, 1.94) | >0.9 | 1.31 (0.84, 2.03) | - | 0.2 | - | 0.67 (0.45, 0.99) | 0.044* | - | 0.93 (0.63, 1.39) | 0.7 |
| Q8b. I am confident I can evaluate extent of ILC disease on mammography (2D with DBT) in women with **dense breasts** | - | 1.42 (0.88, 2.29) | 1.74 (0.93, 3.28) | 0.2 | 1.44 (0.90, 2.30) | - | 0.13 | - | 0.87 (0.57, 1.32) | 0.5 | - | 1.37 (0.90, 2.09) | 0.14 |
| Q9a. I feel additional imaging beyond mammography/DBT is needed to evaluate the extent of ILC disease in women with **non-dense breasts** | - | 0.96 (0.60, 1.52) | 1.14 (0.62, 2.10) | 0.8 | 1.05 (0.67, 1.64) | - | 0.8 | - | 1.27 (0.85, 1.92) | 0.2 | - | 1.77 (1.17, 2.69) | 0.007* |
| Q9b. I feel additional imaging beyond mammography/DBT is needed to evaluate the extent of ILC disease in women with **dense breasts** | - | 1.21 (0.71, 2.05) | 1.21 (0.61, 2.48) | 0.8 | 0.92 (0.54, 1.55) | - | 0.8 | - | 1.42 (0.89, 2.28) | 0.14 | - | 1.17 (0.73, 1.88) | 0.5 |
| Q10a. I routinely recommend MRI to evaluate the extent of ILC disease in women with **non-dense breasts** | - | 1.28 (0.80, 2.04) | 1.09 (0.59, 2.06) | 0.6 | 1.33 (0.84, 2.10) | - | 0.2 | - | 0.83 (0.55, 1.25) | 0.4 | - | 1.72 (1.13, 2.61) | 0.011* |
| Q10b. I routinely recommend MRI to evaluate the extent of ILC disease in women with **dense breasts** | - | 1.11 (0.61, 2.02) | 1.09 (0.50, 2.49) | >0.9 | 1.81 (1.02, 3.15) | - | 0.041* | - | 0.57 (0.33, 0.97) | 0.04* | - | 1.69 (0.99, 2.89) | 0.056* |

Abbreviations: CI, confidence interval; DBT, digital breast tomosynthesis; ILC, invasive lobular carcinoma; OR, odds ratio.

*Statistically significant.

^a^Reference standard.
